# Early Risk Stratification for 30-Day Mortality After In-Hospital Cardiac Arrest: SHAP Interpretable CatBoost Model with m-NUTRIC and Micronutrient Biomarkers

**DOI:** 10.3390/jcm15062310

**Published:** 2026-03-18

**Authors:** Gülseren Elay, Aytaç Güven

**Affiliations:** 1 Department of Internal Medicine, Faculty of Medicine, Gaziantep University, Gaziantep 27310, Turkey; 2 Department of Civil Engineering, Faculty of Engineering, Gaziantep University, Gaziantep 27310, Turkey; aytacguven77@gmail.com

**Keywords:** in-hospital cardiac arrest, 30-day mortality, CatBoost, m-NUTRIC, SHAP, machine learning, risk stratification

## Abstract

**Background/Objectives**: Predicting 30-day mortality after in-hospital cardiac arrest (IHCA) remains challenging. We developed an interpretable CatBoost model that incorporates the m-NUTRIC score, age, and selected micronutrient biomarkers (i.e., magnesium, zinc, vitamin D, and vitamin B12). We compared its performance with that of logistic regression and quantified variable contributions using SHAP. **Methods**: Variables were extracted from the electronic medical records of 880 patients with IHCA admitted to a medical intensive care unit. The CatBoost and logistic regression models were trained on a stratified 80/20 split. The decision threshold was optimized using the Youden index (0.482). Discrimination (ROC-AUC with bootstrap confidence intervals), classification metrics, precision–recall analysis, calibration, and decision curve analysis were reported. **Results**: CatBoost achieved a ROC-AUC of 0.850 (95% confidence interval [CI]: 0.822–0.879) in the training set and 0.827 (95% CI: 0.760–0.887) in the internal test set, outperforming logistic regression (0.797; 95% CI: 0.720–0.861). The test set accuracy, precision, recall, F1-score, specificity, and average precision were 0.761, 0.847, 0.790, 0.817, 0.702, and 0.909, respectively. The Brier score was 0.186. Decision curve analysis showed net benefit across threshold probabilities of 0.20–0.70. The SHAP analysis identified m-NUTRIC and age as the dominant predictors, whereas micronutrients served as complementary contextual factors. **Conclusions**: The CatBoost model consistently outperformed the logistic regression and warrants prospective multicenter validation.

## 1. Introduction

Predicting outcomes after IHCA is clinically challenging [1]. In contemporary cohorts, survival to hospital discharge after adult IHCA is approximately 25% [2]. Many prognostic models rely on logistic regression, which may not capture nonlinear relationships [3,4]. Consequently, risk classification may be imprecise, potentially affecting clinical decisions and resource allocation [5].

Machine learning methods can model complex relationships among multiple variables [6]; however, some approaches, particularly deep neural networks, function as black boxes in which the contribution of individual variables is not readily interpretable [7]. CatBoost, a gradient boosting algorithm, offers several advantages for clinical datasets, including native categorical data handling and built-in regularization to reduce overfitting [8]. The SHapley Additive ExPlanations (SHAP) framework improves model interpretability by quantifying the contribution of each variable’s direction and magnitude to individual predictions [9].

Established severity scores, such as the Acute Physiology and Chronic Health Evaluation II (APACHE II) and the Sequential Organ Failure Assessment (SOFA), are designed to predict overall mortality and organ dysfunction risk but do not directly incorporate nutritional risk. In contrast, the modified Nutrition Risk in the Critically Ill (m-NUTRIC) score is a validated tool for assessing nutritional risk in patients with critical illness, and higher m-NUTRIC values have been consistently associated with increased mortality [10]. Although an association between nutritional risk and outcomes in patients with post-cardiac arrest has been reported, evidence on the independent prognostic contribution of m-NUTRIC, specifically in IHCA, remains limited [11,12]. Furthermore, micronutrient imbalances during the post-resuscitation period may have additional prognostic value, and integrating nutritional risk with metabolic markers could improve predictive models [13,14,15].

This study aimed to develop an interpretable CatBoost model that uses the m-NUTRIC score, age, and selected micronutrient biomarkers (magnesium, zinc, vitamin D, and vitamin B12) to predict 30-day mortality after IHCA, compare its performance with a logistic regression baseline, and quantify variable contributions using SHAP.

## 2. Materials and Methods

### 2.1. Ethical Approval

The study was approved by the Ethics Committee of Gaziantep Islamic Science and Technology University (Approval No: 2025-Two ONP 0074) on 24 October 2025. This study was retrospective, and no identifying data were used. The ethics committee waived the requirement for informed consent.

### 2.2. Study Design and Patient Population

This retrospective cohort study included 883 patients who developed IHCA, achieved return of spontaneous circulation (ROSC), and were admitted to the MICU between October 2019 and October 2022. IHCA events occurred outside the ICU in this cohort. Demographic information, comorbidities, and cause of IHCA were obtained from the electronic medical record. The index time was defined as the time of admission to the MICU. Biochemical measurements were obtained from blood samples collected within the first 24 h after admission to the MICU. The APACHE II, Charlson Comorbidity Index (CCI), m-NUTRIC, and Glasgow Coma Scale (GCS) scores were obtained from the EMR records. The APACHE II, CCI, and GCS scores were recorded for descriptive purposes; they were not included in the CatBoost mortality model inputs. The length of ICU stay was obtained from the EMR and reported only in descriptive analyses; it was not included in the predictive model or SHAP analyses.

### 2.3. Inclusion and Exclusion Criteria

Inclusion criteria:

Age ≥ 18 years;

In-hospital cardiac arrest outside the intensive care unit;

The return of spontaneous circulation (ROSC) achieved;

Admission to the medical intensive care unit (MICU) after IHCA;

Survival ≥ 6 h after ROSC, allowing evaluation of early post-resuscitation clinical and laboratory data.

Exclusion criteria:

Out-of-hospital cardiac arrest;

IHCA occurring during the ICU stay;

History of prior IHCA during the same period of hospitalization;

Unwitnessed or recurrent cardiac arrest;

Cardiac arrest in the operating room or in the post-anesthesia care unit;

CPR duration < 2 min, considered indicative of rapidly reversible events or documentation artifacts rather than sustained cardiac arrest;

Uncertain or undocumented CPR duration or start time;

Delay ≥ 7 min from documented cardiac arrest recognition to initiate chest compressions, based on timestamps in electronic medical records;

Admission to the ICU > 24 h after ED evaluation;

Confirmation of SARS-CoV-2 infection.

Although the Cardiac Arrest-Specific Prognostic Index (CASPRI) was calculated for descriptive purposes, it was not used as an exclusion criterion or as an input to the machine learning model to avoid incorporation bias.

**Figure 1:**
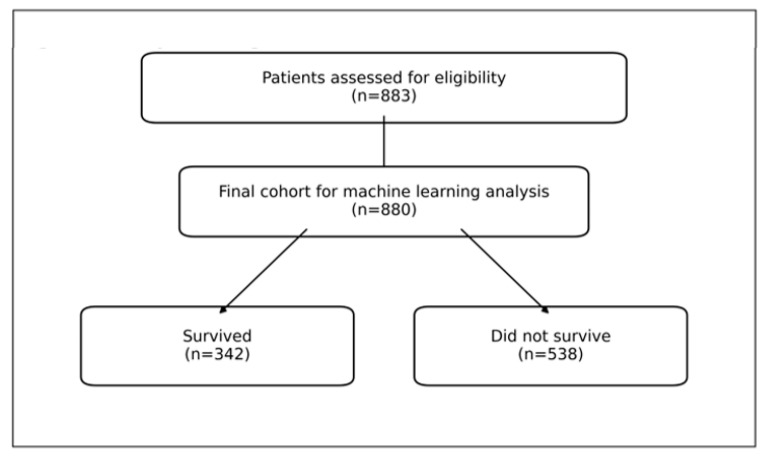
Patient selection and exclusion criteria.

To minimize missing data and early post-arrest heterogeneity and ensure that clinical and laboratory data from the first 24 h were evaluable, ROSC within 20 min and survival for at least 6 h after ROSC were required (Figure 1).

### 2.4. Resuscitation Protocol

All IHCA events were managed in accordance with the European Resuscitation Council (ERC) ALS guidelines applicable during the study period. DNR orders were not routinely implemented or documented as a standard practice at the institution during the study period.

### 2.5. Endpoint

The primary endpoint was all-cause mortality within 30 days of IHCA. Thirty-day mortality was ascertained from EMR and follow-up records.

### 2.6. Statistical and Software Analyses

The Statistical Package for the Social Sciences 22.0 was used for descriptive statistics and group comparisons. The Shapiro–Wilk test was used to assess normality. Non-normally distributed continuous variables were compared using the Mann–Whitney U test or the Kruskal–Wallis test. The categorical variables were compared using the chi-square test. Spearman’s correlation coefficients were calculated for continuous variables. A two-sided *p* < 0.05 was considered statistically significant. The machine learning model was developed and evaluated using Python Statistical analyses were performed using SPSS version 22.0 (IBM Corp., Armonk, NY, USA). Machine learning analyses were conducted using Python 3.9 (Python Software Foundation, Wilmington, DE, USA) with the CatBoost 1.2, scikit-learn 1.3, and SHAP 0.43 libraries.

### 2.7. Management of Missing Data

Three out of 883 patients (0.3%) had missing m-NUTRIC predictor data and were excluded from the ML analysis. No imputation was performed. Model training and evaluation were conducted in 880 complete cases. Missingness was not associated with 30-day mortality. Given the very low proportion of missing data, complete-case analysis was unlikely to introduce selection bias. The baseline descriptive characteristics of the full cohort (*n* = 883) were reported.

### 2.8. Machine Learning Model and Internal Evaluation

The CatBoost algorithm was used to predict the 30-day mortality. The complete-case dataset (*n* = 880) was randomly split into a training set (80%, *n* = 704; 419 deaths, 285 survivors) and an internal test set (20%, *n* = 176; 119 deaths, 57 survivors) using stratified sampling to preserve the class distribution. The random seed was fixed (seed = 42) to ensure reproducibility, and no repeated random splits were performed. No data preprocessing or feature scaling was applied, consistent with the tree-based structure of the CatBoost algorithm.

Hyperparameter optimization was performed exclusively in the training set using stratified 5-fold cross-validation, with the ROC-AUC as the primary optimization metric. The internal test set was not used for hyperparameter tuning or model selection. The final hyperparameters were: tree depth = 5, learning rate = 0.05, column sampling rate = 0.7, and L2 regularization. Early stopping was applied within the training set to mitigate overfitting.

### 2.9. Model Inputs, Decision Thresholds, and Performance Criteria

The model inputs were age, m-NUTRIC, magnesium, zinc, vitamin B12, and vitamin D, all of which were measured within the first 24 h after MICU admission. Post-baseline variables were not included. The positive class was defined as “dead.” The optimal decision threshold was determined in the training set by maximizing the Youden index (J = sensitivity + specificity − 1), yielding a threshold of 0.482, which was applied unchanged to the internal test set. The Youden index was selected for its simplicity, objectivity, and independence from disease prevalence. It is widely used in binary classification for medical decision-making.

The receiver operating characteristic curve (ROC-AUC), accuracy, precision, recall, F1-score, and specificity were used to assess performance. Additionally, precision–recall analysis with AP and decision curve analysis (DCA) were reported for the internal test set. Precision–recall analysis was included as a complement to ROC-AUC given class imbalance. For DCA, the net benefit was calculated across threshold probabilities of 0.01–0.80 using probability-based model outputs and compared with “treat-all” and “treat-none” strategies. The DCA settings are detailed in the Supplementary Materials. Calibration was evaluated using the Brier score and a calibration curve, which was constructed by dividing the predicted probabilities into 10 decile bins of equal size and comparing the mean predicted risk per bin with the observed event rate.

### 2.10. Baseline Model: Logistic Regression Analysis (LRA)

A logistic regression model was trained using the same six predictors as a comparative baseline. Balanced class weights were applied to account for the imbalanced outcome distribution, and all input features were standardized before training. The Youden index in the training set (threshold = 0.485) was used to determine the optimal threshold, which was applied unchanged to the internal test set. This comparison allowed a direct assessment of whether the additional complexity of gradient boosting provides meaningful improvements over logistic regression.

### 2.11. ROC-AUC and 95% CI

For both models, 95% confidence intervals for ROC-AUC were calculated separately for the training and internal test sets using 2000 stratified nonparametric bootstrap resamples. Confidence intervals were derived from the bootstrap distribution’s 2.5th and 97.5th percentiles.

### 2.12. Model Explainability

The model interpretability was assessed using SHAP. The global feature importance was quantified by mean absolute SHAP values, and the SHAP dependence plots were used to visualize the variable effects. The SHAP values were derived from the final CatBoost model. The SHAP analyses excluded post-baseline variables.

## 3. Results

### 3.1. Characteristics of the Study Population

A total of 883 patients who met the inclusion criteria were included in the descriptive analyses. Model performance was evaluated in 880 patients with complete predictor data. The mean age was 64.84 ± 16.93 years, and 55.5% were male. The 30-day all-cause mortality rate was 59.6%. The mean m-NUTRIC score was 5.19 ± 1.86. The baseline demographic and clinical characteristics are summarized in Table 1.

### 3.2. Performance of the CatBoost Model and Comparison with the Baseline Mortality Model (Internal Evaluation)

**Figure 2:**
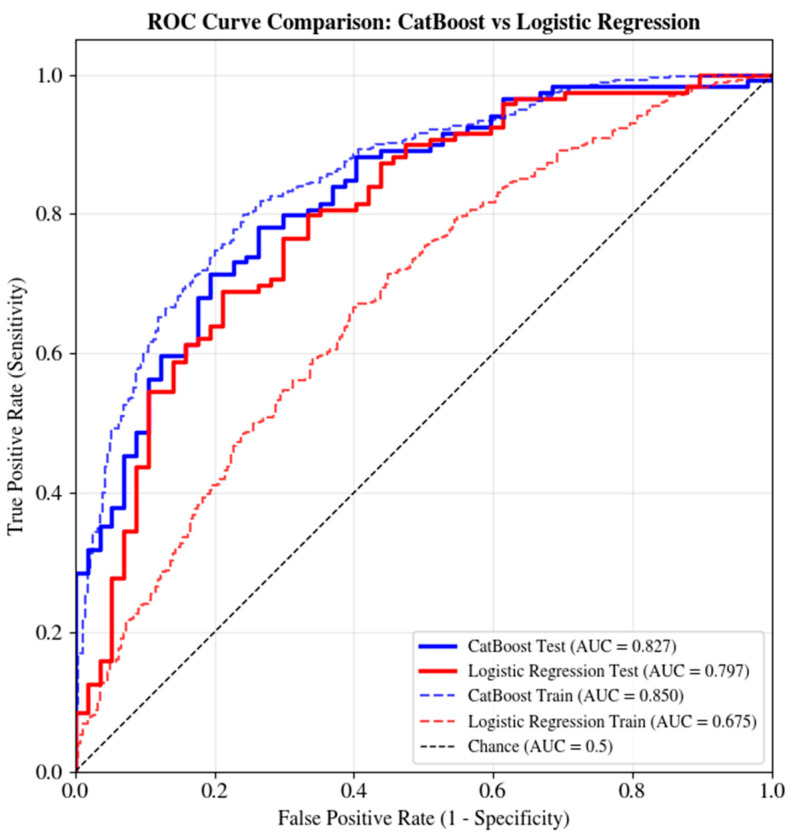
ROC curves for 30-day mortality prediction in the training and internal test sets for CatBoost and logistic regression models.

The discriminative capacity of both the CatBoost model and the logistic regression baseline was evaluated using receiver operating characteristic (ROC) analysis. The receiver operating characteristic (ROC) curve plots sensitivity (true positive rate) against 1 − specificity (false positive rate) across all classification thresholds, assessing the model’s ability to distinguish between patients who died and those who survived. The area under the curve (AUC) is a summary measure of discrimination, with values ranging from 0.5 (no discrimination) to 1.0 (perfect discrimination). In clinical prognostic modeling, AUC values of 0.70–0.80, 0.80–0.90, and >0.90 are generally considered acceptable, excellent, and outstanding, respectively. Figure 2 presents the receiver operating characteristic (ROC) curves for both models in the training and test sets, accompanied by 95% confidence intervals derived through stratified nonparametric bootstrap resampling. Bootstrap resampling accounts for sampling variability while preserving each resample’s class distribution.

In the training set, the CatBoost model achieved a ROC-AUC of 0.850 (95% CI: 0.822–0.879), indicating excellent discrimination. In the internal test set (*n* = 176), the ROC-AUC was 0.827 (95% CI: 0.760–0.887), indicating good discrimination in unseen data. The relatively modest train–test performance gap (ΔAUC = 0.023) suggests that the regularization strategy and conservative model complexity effectively limited overfitting. This stability is important for clinical deployment, where a consistent performance across different patient populations is essential.

The ROC curve for CatBoost remains well above the diagonal reference line throughout its trajectory, confirming that all classification thresholds are discriminated consistently.

The logistic regression model with balanced class weights revealed a different pattern. In the training set, logistic regression achieved a ROC-AUC of 0.675 (95% CI: 0.635–0.716), which was substantially lower than that of CatBoost. In the test set, the ROC-AUC was 0.797 (95% CI: 0.720–0.861). Logistic regression exhibited a paradoxical pattern, with the test performance exceeding the training performance (ΔAUC = −0.122). This finding suggests that logistic regression’s linear assumption may lack flexibility to capture complex relationships in the training data, possibly reflecting underfitting. The improved test performance may reflect that the test set, by chance, contained cases where the linear approximation was more adequate, although this pattern would be unlikely to replicate across external validation cohorts.

In the test set, CatBoost outperformed the logistic regression, with an AUC improvement of +0.030 (0.827 vs. 0.797). This improvement may be clinically meaningful. Although the 95% confidence intervals for both models overlap (CatBoost: 0.760–0.887; LR: 0.720–0.861), the difference does not reach statistical significance at α = 0.05 given the sample size. However, the consistent advantage of CatBoost across multiple metrics supports its selection as the preferred model.

An AUC of 0.827 in the test set indicates that when a randomly selected patient who died is compared with a randomly selected survivor, the CatBoost model assigns a higher mortality probability to the deceased patient approximately 83% of the time, compared with 80% for logistic regression.

Although receiver operating characteristic (ROC) analysis provides a threshold-independent evaluation of discrimination, practical clinical application requires the conversion of probability estimates into binary classifications using a fixed decision threshold. Applying the training-derived thresholds (P(Dead) ≥ 0.482 for CatBoost and P(Dead) ≥ 0.485 for logistic regression), confusion matrices were computed for both models in both the training and test sets. Despite the different model architectures, the optimal thresholds were very similar: 0.482 for CatBoost and 0.485 for logistic regression. Then, these thresholds were fixed and applied to the test set for the final performance evaluation.

**Figure 3:**
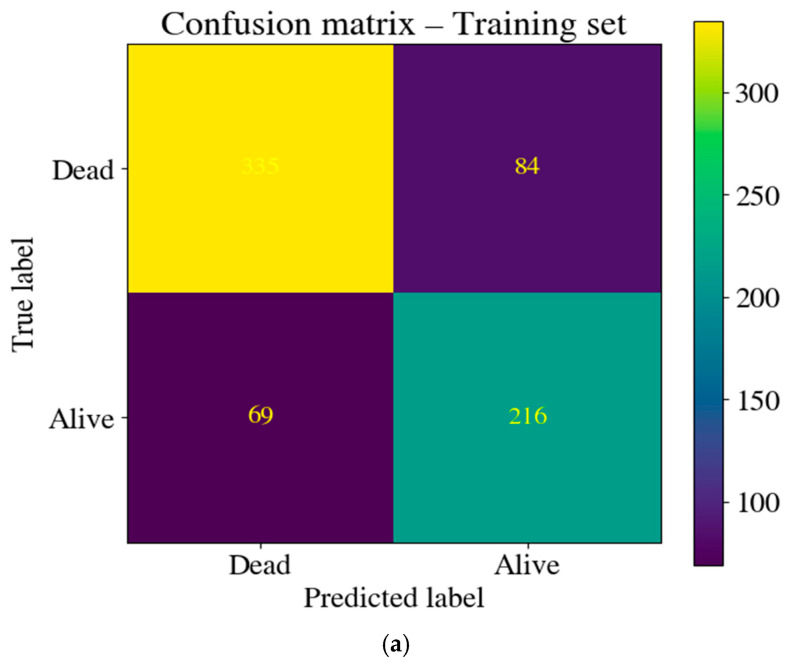

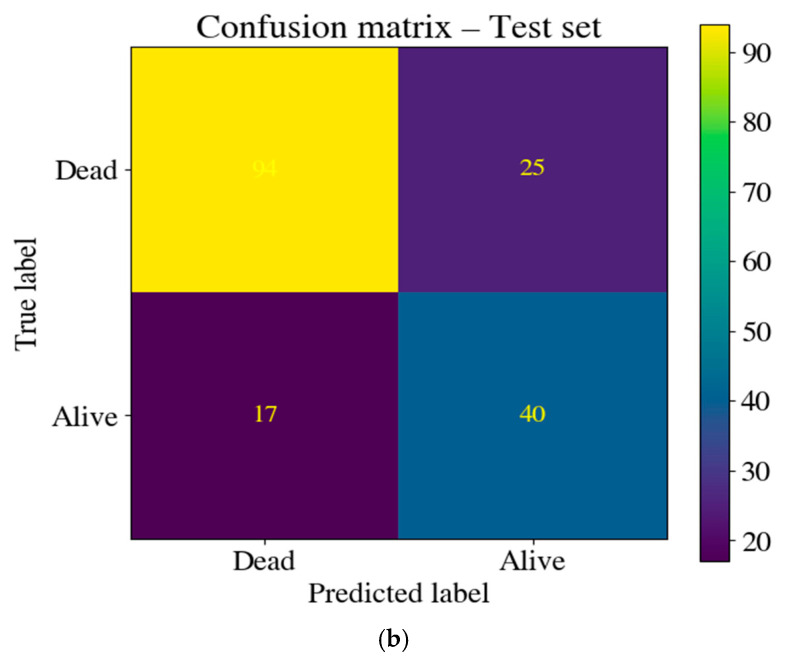
(**a**) Confusion matrix for the training set at the selected threshold of the CatBoost model. (**b**) Confusion matrix for the internal test set at the selected threshold of the CatBoost model.

Figure 3a and Figure 3b present the training and test set confusion matrices for the CatBoost model, respectively. The overall classification accuracy was 0.783 in the training set and 0.761 in the test set at the selected threshold. The minimal decrease in accuracy from the training to the test set further supports the model’s generalizability. In the training set (Figure 3a), 335 of 419 deaths were correctly identified (sensitivity = 79.9%), whereas 84 deaths were misclassified as survivors. Of the 285 survivors, 216 were correctly identified (specificity = 75.8%), while 69 were misclassified as deaths. The test set (Figure 3b) showed a comparable performance: 94 of 119 deaths were correctly identified (sensitivity = 79.0%), and 40 of 57 survivors were correctly identified (specificity = 70.2%).

**Figure 4:**
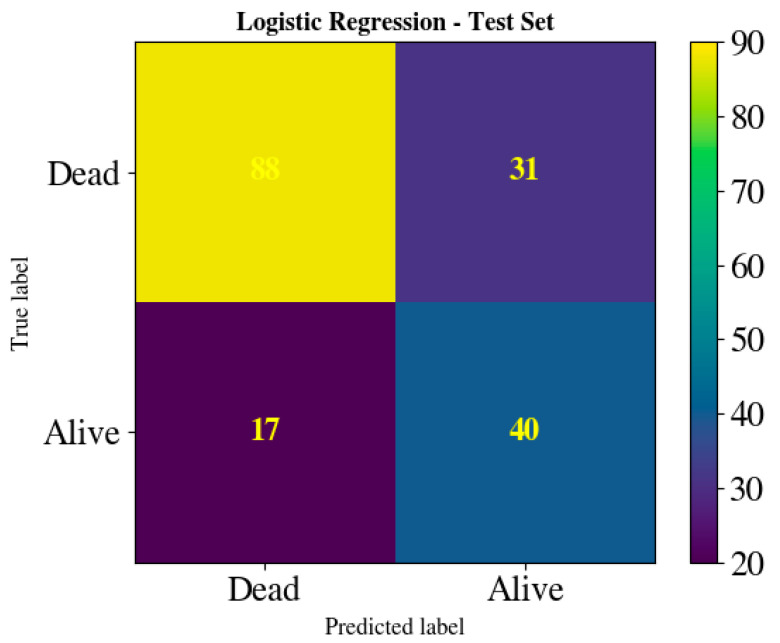
Confusion matrix for the internal test set at the selected threshold for the logistic regression model.

Figure 4 shows the corresponding confusion matrix for logistic regression. The overall classification accuracy was 0.727 (128/176 correct classifications) at the optimal threshold of 0.485. The model correctly classified 88 of 119 mortality cases (true positives), corresponding to a sensitivity of 73.9%. The specificity was 70.2% (40/57 survivors were correctly identified), which was identical to that of CatBoost. Logistic regression produced 31 false negatives (26.1% of actual deaths) and 17 false positives (29.8% of actual survivors). The higher false-negative count compared with CatBoost (31 vs. 25) represents a notable limitation.

CatBoost achieved superior accuracy (+0.034) primarily through improved mortality class sensitivity. Both models achieved identical specificity (70.2%), but CatBoost detected six additional deaths that logistic regression missed (94 vs. 88 true positives). This improvement in mortality detection is clinically relevant for an early warning instrument, as failure to identify high-risk patients carries greater consequences than incorrectly flagging low-risk patients for additional monitoring.

Because overall accuracy can be misleading with class imbalance, we computed class-specific performance metrics (precision, recall, and F1-score) for both outcome classes. Table 2 presents these metrics for the training and test sets. For the mortality class (Dead), the test set achieved a precision of 0.847, recall of 0.790, and F1-score of 0.817. For the survivor class (Alive), the test set achieved a precision of 0.615, recall of 0.702, and F1-score of 0.656. The model performed better for the mortality class (F1-score: 0.817) than for survivors (0.656). This asymmetry reflects both the class distribution (higher prevalence of deaths) and the inherent trade-off when a single threshold is used. From a clinical perspective, this pattern is appropriate for an EWI, where missing high-risk patients carries greater consequences than false alarms. The high precision (0.847) ensures that the flagged patients are at an elevated risk.

Class-specific performance metrics were also computed for both models on the internal test set. Table 3 presents these metrics. CatBoost achieved a precision of 0.847, recall of 0.790, and F1-score of 0.817 for the mortality class (dead). Logistic regression achieved a lower performance: precision of 0.838, recall of 0.739, and F1-score of 0.786. CatBoost’s F1-score improvement of +0.031 reflects its superior ability to balance precision and recall for mortality prediction. For the survivor class (Alive), CatBoost achieved an F1-score of 0.656 compared with 0.625 for the logistic regression (+0.031 improvement). The consistent improvement across both classes indicates that CatBoost is not limited to a single outcome category.

CatBoost outperformed the logistic regression across all metrics (F1-score: 0.817 vs. 0.786), with higher sensitivity for the mortality class while maintaining identical specificity. Table 4 summarizes the comparison of CatBoost and logistic regression for the internal test set.

Comparison with the logistic regression baseline demonstrated that CatBoost provided consistent improvements across all discrimination and classification metrics. Although the ROC-AUC confidence intervals overlap, the consistent advantage across multiple metrics—including accuracy (+0.034), sensitivity (+0.051), and F1-scores (+0.031 for both classes)—supports the selection of CatBoost as the preferred model. The gradient boosting architecture enabled CatBoost to capture nonlinear relationships and variable interactions that logistic regression may miss, contributing to the observed performance differences.

### 3.3. Feature Contribution and SHAP Analysis

**Figure 5:**
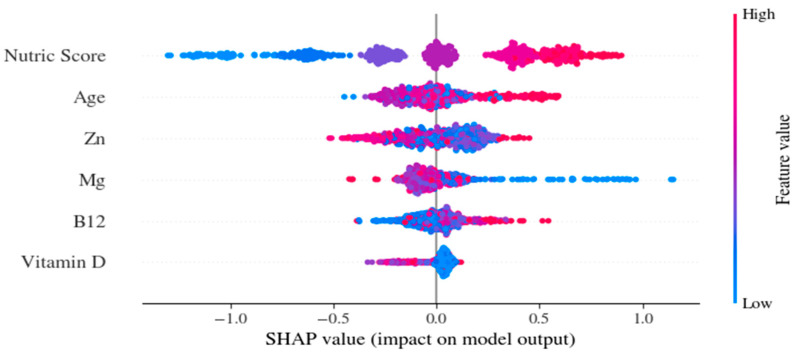
SHAP summary plot showing the variables’ contribution to the CatBoost model prediction.

In the SHAP analysis, the m-NUTRIC score, followed by age, had the highest contribution to the model outputs (Figure 5).

Model features were selected based on clinical relevance and hypotheses from the literature. The m-NUTRIC score is a validated index that is widely used to quantify nutritional risk of patients with critical illness. The selected micronutrients—magnesium, zinc, vitamin B12, and vitamin D—were included because of their roles in immune regulation and cardiovascular stability and their association with mortality in patients with critical illness.

Age was incorporated as a separate feature not only because it is an independent predictor of mortality but also because it reflects physiologic reserve beyond what is captured by scoring systems. Although age is a component of the m-NUTRIC score, both variance inflation factor (VIF) and SHAP interaction analyses were used to evaluate potential collinearity. These analyses did not reveal any problematic redundancy. The SHAP plots further demonstrate that age provides distinct predictive value and modulates risk patterns independent of m-NUTRIC.

The prognostic impact of micronutrient status in patients with cardiac arrest remains limited. This study aimed to address this gap using an exploratory, hypothesis-generating approach. Given the rapidly aging global population, these features are increasingly relevant for risk stratification in in-hospital cardiac arrest.

The micronutrient markers (Mg, Zn, vitamin D, and vitamin B12) contributed less to SHAP than m-NUTRIC and age (Figure 5). For example, the SHAP dependence plot for zinc (Supplementary Figure S4) shows that lower zinc levels are associated with increased predicted mortality risk, with the effect appearing more pronounced in older patients. The patterns and possible nonlinear effects of the predictor variables are detailed in the SHAP dependence plots in Supplementary Figures S2–S4.

### 3.4. Precision–Recall Analysis (Internal Test Set)

Given the class imbalance in the outcome distribution (61.1% mortality rate), the ROC analysis was supplemented with the PR analysis. PR analysis focuses on the positive class (mortality) without being influenced by true negatives that can inflate the ROC-AUC, making it more informative for imbalanced datasets.

**Figure 6:**
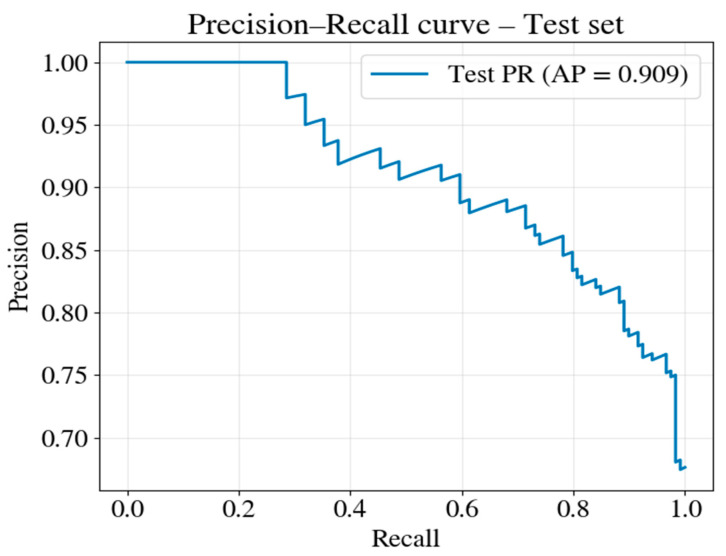
Precision–recall curve for the mortality class of the CatBoost model in the internal test set.

Figure 6 shows the PR curve of the test set. The model achieved an average precision (PR-AUC) of 0.909. This value substantially exceeds the baseline AP of approximately 0.68 (class prevalence), confirming that the model provides meaningful risk stratification beyond the prevalence-based prediction.

The PR curve shape reveals important operational characteristics. At low recall values (left side of the curve), precision approaches 1.0, indicating that the model’s highest-confidence mortality predictions are highly reliable—patients assigned the highest predicted probabilities almost invariably die. As recall increases, precision gradually declines but remains above 0.80 until approximately 85% of deaths are captured. This indicates that the model can identify most high-risk patients while maintaining an acceptable PPR—a key property for clinical decision support.

### 3.5. Probability Calibration and the Brier Score (Internal Test Set)

Beyond discrimination, calibration assesses whether the predicted probabilities accurately reflect the frequency of the observed event. In a well-calibrated model, approximately 70% of patients assigned a 70% predicted mortality probability actually die. Calibration is essential for clinical decision support applications in which probability estimates inform treatment intensity, resource allocation, or prognostic discussions with patients and families.

**Figure 7:**
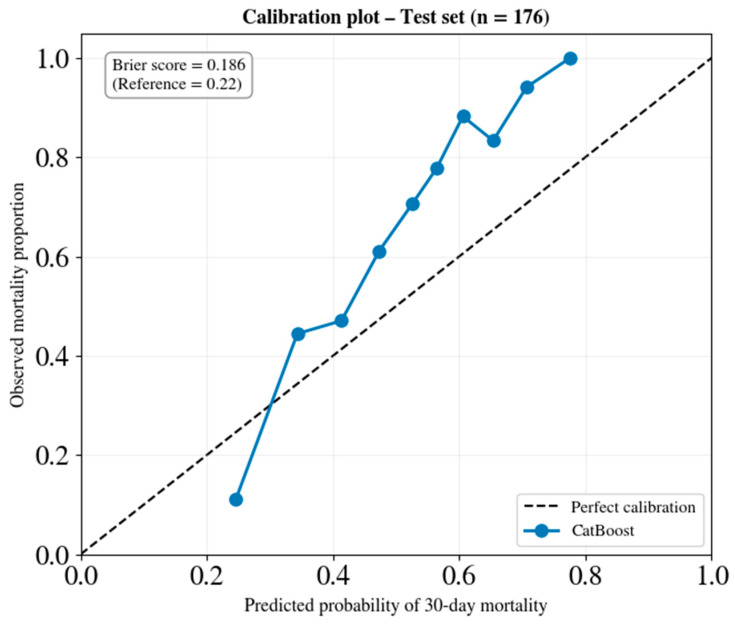
Calibration plot of the CatBoost model in the internal test set.

Figure 7 presents the calibration plot for the internal test set, which was constructed using 10 decile bins of equal size. For each bin, the mean predicted probability of in-hospital mortality (x-axis) is plotted against the observed mortality proportion (y-axis). The dashed diagonal line represents a perfect calibration, where the predicted probabilities exactly match the observed outcomes. Points falling above this line indicate underestimation of risk, while points below indicate overestimation.

The Brier score for the internal test set was 0.186. The Brier score ranges from 0 (perfect) to 1 (worst prediction). A non-informative model predicting the marginal prevalence (0.68) for all patients would achieve a Brier score of approximately 0.22. The observed score of 0.186 represents a 15% improvement over the baseline, indicating that the model’s probability estimates convey meaningful prognostic information.

Visual inspection of the calibration plot (Figure 7) reveals reasonable alignment with the identity line across the clinically relevant intermediate-probability range (approximately 0.3–0.7), where most clinical decisions are made. In this range, predicted probabilities correspond closely to observed mortality rates, supporting the interpretation of model outputs as approximate absolute risk estimates suitable for individual patient counseling and treatment planning. Some deviation from perfect calibration is observed at the distribution extremes (predicted probabilities below 0.3 and above 0.8), which is expected given the small number of patients in these bins.

These findings have direct implications for bedside decision-making. A model prediction of 60% mortality probability can be interpreted at face value—approximately 60% of patients with similar predicted probabilities died. This enables meaningful prognostic communication and supports shared decision-making regarding treatment intensity and resource allocation.

### 3.6. Decision Curve Analysis

Decision curve analysis was performed to evaluate the potential clinical utility of the predictive model. Unlike standard discrimination metrics, DCA explicitly incorporates the clinical consequences of prediction errors by calculating “net benefit” across a range of threshold probabilities. The net benefit weighs the benefits of correctly identifying high-risk patients against the costs of false-positive classifications, weighted according to the clinical decision threshold. The threshold probability represents the mortality risk level at which a clinician would change management—for example, initiating aggressive nutritional support, intensifying monitoring, or discussing care goals with the family). Lower thresholds (e.g., 0.30) favor early detection at the cost of more false positives, whereas higher thresholds (e.g., 0.60) reflect a more conservative approach, reserving intervention for patients with substantially elevated risk. For each threshold probability between 0.01 and 0.80, net benefit was calculated for three strategies: (1) the CatBoost prediction model, (2) treating all patients as high-risk (“treat-all”), (3) treating no patients as high-risk (“treat-none”). These reference strategies represent the clinical practice’s extremes and provide context for interpreting model performance.

**Figure 8:**
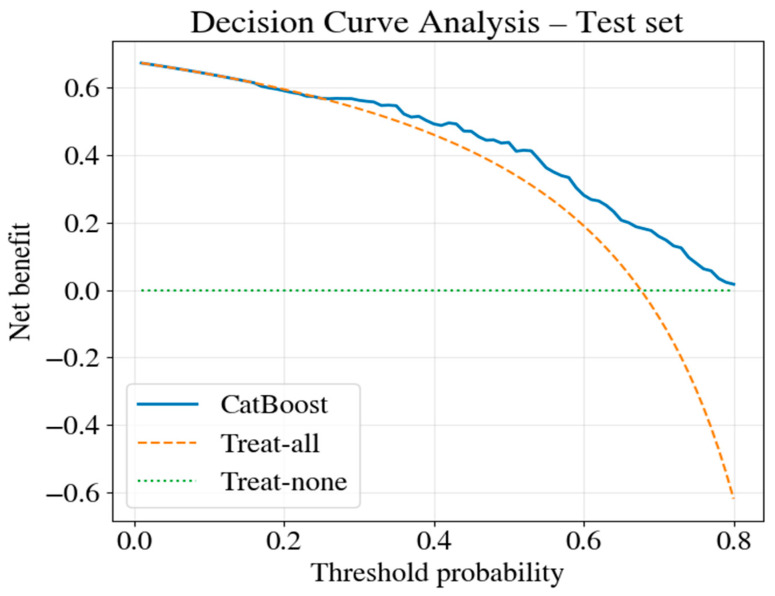
Decision curve analysis of the CatBoost model in the internal test set.

Figure 8 shows that, across a broad interval of clinically plausible risk thresholds (approximately 0.20–0.70), the CatBoost model yielded higher net benefit than both the treat-all and treat-none strategies. The vertical distance between the model curve and the reference strategies at any given threshold represents the model’s net improvement in clinical outcomes.

Practical interpretation of the threshold range (0.20–0.70): this range encompasses the most realistic clinical scenarios in intensive care unit settings.

At a threshold of 0.20 (20%): The clinician would intervene for any patient with ≥20% predicted mortality risk. This represents an aggressive, resource-intensive approach that is appropriate for settings where the intervention cost is low relative to the benefit of early identification. The model provides a net benefit over treating everyone.

At a threshold of 0.40 (40%): A moderate threshold at which the clinician balances sensitivity and resource use. The model achieves substantial net benefit by correctly identifying high-risk patients and avoiding unnecessary interventions in lower-risk individuals.

At threshold 0.60 (60%): A conservative threshold appropriate for interventions with higher costs or risks, at which the clinician requires stronger evidence of elevated mortality risk before changing management. The model maintains a positive net benefit over alternative strategies even at this threshold.

The treat-all strategy remains competitive at threshold probabilities below 0.20 because the low threshold implies that clinicians would treat nearly all patients regardless of predicted risk, making model-based selection unnecessary. All strategies converge toward zero net benefit at threshold probabilities above 0.70 because few patients exceed such stringent risk criteria, and predictive modeling becomes less dependent on clinical decisions.

The decision curve analysis supports the use of the CatBoost model to guide patient selection for intensified monitoring, nutritional interventions, or care escalation. On average, the implementation of the model would improve clinical outcomes compared with universal treatment or no risk-based selection. The model’s utility across the threshold range of 0.20–0.70 indicates robustness to varying clinical preferences and institutional resource constraints.

### 3.7. Summary of the CatBoost Model Performance

Table 5 summarizes all model performance metrics for direct comparison between training and test set results.

Consistent results were observed between the training and test sets across all major metrics. The performance gap between the training and test sets was minimal for discrimination metrics (ΔAUC = 0.023, ΔAccuracy = 0.022), indicating effective regularization and limited overfitting. The mortality class F1-score was stable between the training (0.814) and test (0.817) sets, with the test set value slightly exceeding the training value—an unusual pattern that reinforces confidence in the model’s generalizability. The test set PR-AUC of 0.909 and Brier score of 0.186 indicate a strong predictive performance that substantially exceeds chance-level baselines.

## 4. Discussion

This study demonstrated that an ML model developed with the CatBoost algorithm can classify the 30-day mortality risk after in-hospital cardiac arrest, providing good discrimination in the training set and moderate-to-good discrimination in the internal test set. The performance decline from training (ROC-AUC 0.850) to internal testing (ROC-AUC 0.827) suggests potential model optimism, and the observed development performance may overestimate real-world performance. Therefore, this model should be interpreted as exploratory and hypothesis-generating rather than ready for clinical application. Further validation in prospective and multicenter cohorts is required before clinical use is considered.

The internal validation strategy and sampling variability may also influence the performance decline [16]. Although mitigation strategies, such as early stopping and L2 regularization, have been applied [17], the current split-based internal validation provides a limited estimate of model stability. Therefore, performance metrics should be cautiously interpreted. No repeated cross-validation or bootstrapped model selection was performed. Future work should explore these more rigorous internal validation strategies to provide better estimates of the variability in expected performance. To demonstrate generalizability, external validation following current reporting guidelines is necessary [16,18].

Traditional prognostic approaches typically assume linear relationships among age, m-NUTRIC, and mortality [3,10]. The SHAP analyses showed that m-NUTRIC and age were the top-ranked contributors to predicted risk, highlighting their joint nonlinear influence rather than suggesting direct causality. SHAP values reflect learned associations and should not be interpreted as causal evidence.

The literature suggests that fixed thresholds for m-NUTRIC may not perform consistently across different populations and that it may be more appropriate to interpret this variable within a multivariate framework rather than evaluate it as a stand-alone decision tool [19]. Our findings suggest that prognostic information can be combined more effectively when these variables are integrated into a multivariate framework, and gradient boosting approaches can better capture such complex relationships [20]. Although the ROC-AUC value remains moderate in the internal test set, reporting PRP provides a more appropriate evaluation framework for imbalanced clinical datasets [21].

Accuracy and precision can be artificially inflated in imbalanced datasets despite a poor minority class performance. To address this issue, we report the AP and PR-AUC, which more accurately reflect the model’s effectiveness in predicting the clinically relevant outcome—30-day mortality. Therefore, we did not rely solely on ROC-based reporting but also used metrics that are more sensitive to class imbalance, such as average precision, for a more balanced assessment of positive class performance.

Exploratory analyses were conducted using an external cohort to illustrate the potential challenges in model transferability. Because of limited variable overlap and significant class imbalance, technical details and performance findings are reported in the Supplementary Materials and should not be interpreted as external validation.

Although the external dataset was analyzed purely for illustrative purposes, reporting high accuracy and precision in a class-imbalanced cohort may create a misleading impression for readers unfamiliar with the limitations of these metrics. This concern is acknowledged to prevent overinterpretation.

SHAP enhances interpretability by identifying which variables contribute to predictions and in which direction, an approach widely used and validated in health research [22].

The systematic comparison between CatBoost and logistic regression, trained on identical features, directly addresses whether ML approaches provide added value over traditional statistical methods for this clinical prediction task. CatBoost achieved a superior test set AUC (0.827 vs. 0.797, Δ = +0.030), which translates to a potentially meaningful advantage in distinguishing high-risk from low-risk patients, although the confidence intervals overlap. CatBoost also achieved a higher classification accuracy (0.761 vs. 0.727, Δ = +0.034), primarily driven by improved sensitivity for the mortality class (0.790 vs. 0.739, Δ = +0.051); this sensitivity improvement was achieved without sacrificing specificity (both models: 0.702). F1-scores improved consistently across both outcome classes (+0.031 for both Dead and Alive classes), indicating that CatBoost’s advantage is not merely an artifact of threshold selection or class imbalance handling. For generalization stability, CatBoost showed a modest train–test performance gap (ΔAUC = 0.023), suggesting effective regularization, whereas logistic regression showed a paradoxical improvement from training to test (ΔAUC = −0.122), which may reflect limited capacity to capture nonlinear relationships in this dataset.

These findings support the selection of CatBoost, although logistic regression remains viable when linear interpretability is prioritized or computational resources are limited. The modest performance gap suggests that most predictive signals are derived from linear predictor–outcome relationships, with incremental but consistent gains from nonlinear modeling. Future studies may explore ensemble approaches that combine the interpretability of logistic regression with the flexibility of GBMs.

This study illustrates how nonlinear relationships between heterogeneous inputs, such as m-NUTRIC and micronutrient markers, can be modeled and interpreted using gradient boosting and SHAP.

Micronutrients alone are not strong prognostic markers, and conflicting results have been reported. Evaluating micronutrients together with other clinical factors may be more meaningful in revealing their limited but informative contributions [23,24]. In this study, micronutrients should be interpreted as secondary, complementary markers that reflect the patient’s biological context alongside key contributors such as age and m-NUTRIC, and that may modulate risk prediction, rather than serving as strong independent predictors. AI-based methods may help clarify the role of micronutrients in clinical decision support [25]. Decision curve analysis showed that the model provided net benefit over the “treat-all” and “treat-none” strategies within a threshold probability range of approximately 0.20–0.70 in the internal test set [26]. Clinically, this range corresponds to scenarios where clinicians may reasonably consider intensified monitoring or supportive interventions for patients judged to be at intermediate-to-high mortality risk, while avoiding unnecessary intervention in clearly low-risk patients. Thus, the decision curve results suggest the model’s potential clinical usefulness within a realistic range of decision thresholds rather than across all possible risk levels. Performance monitoring and safety frameworks should be re-evaluated as data distributions shift over time [27,28]. The variable performance observed in commercial ML models and the need for external validation require caution in critical settings, such as IHCA [29].

Because this study has a retrospective, single-center design, it may be subject to selection and measurement bias, and its external validity may be limited. These limitations should be considered when interpreting the study findings. The difference between training and internal test performance highlights the importance of prospective and multicenter validation. Additionally, the calibration of the probability estimates should be confirmed in the external cohorts. Because the external cohort findings are reported under limited conditions of variable overlap, they constitute preliminary evidence of portability and should be supported by additional validation; they should not be interpreted as a claim of generalizability.

## 5. Conclusions

This study demonstrated that ML approaches can effectively classify the risk of 30-day mortality after in-hospital cardiac arrest. The CatBoost model achieved a good discriminative performance in both the training (ROC-AUC: 0.850) and internal test sets (ROC-AUC: 0.827) and consistently outperformed the logistic regression baseline across multiple metrics, including accuracy, sensitivity, and F1-score. This systematic comparison confirmed that gradient boosting methods provide added value over traditional linear approaches for this clinical prediction task by capturing nonlinear relationships and variable interactions. SHAP analyses revealed that m-NUTRIC and age were the highest-ranking predictors of risk. Micronutrients, such as magnesium, zinc, vitamin D, and vitamin B12, had small but complementary contributions, rather than acting as strong independent predictors, serving as secondary factors that modulate the effect of key contributors. Therefore, micronutrients alone should not be interpreted as strong prognostic markers; however, they can provide additional context for risk classification within a multivariate framework. The model demonstrated acceptable calibration (Brier score: 0.186) and clinical utility across a broad range of decision thresholds (0.20–0.70), supporting its potential application in identifying high-risk patients who may benefit from intensified monitoring or supportive care. However, considering the retrospective, single-center design and the exploratory nature of this study, prospective multicenter validation is required before clinical implementation. An exploratory portability analysis is reported in the Supplementary Materials.

## Figures and Tables

**Table 1:** Patient demographics and baseline characteristics (*n* = 883 patients).

Variable	Value
Age (years) *	64.84 ± 16.93
Male **	490 (55.5)
Chronic renal disease **	133 (15.1)
Diabetes mellitus **	284 (32.2)
Hypertension **	321 (36.4)
COPD **	55 (6.2)
Cancer **	174 (19.7)
Glasgow Coma Scale score ***	4 (3–5)
APACHE II score *	25 ± 3.16
Charlson Comorbidity Index *	4.5 ± 1.12
Zinc (µg/dL) *	54.85 ± 24.05
Magnesium (mg/dL) ***	2.1 (0.8–2.4)
Vitamin D (ng/mL) ***	10 (0.45–110)
Vitamin B12 (pg/mL) ***	345 (50–1525)
m-NUTRIC score *	5.19 ± 1.86
30-day all-cause mortality rate **	526 (59.6)

* Data are presented as mean ± standard deviation. ** Data are presented as the number (percentage). *** Data are presented as median (minimum–maximum) to reflect the full biological range and clinically relevant extreme values observed in this critically ill population. Abbreviations: APACHE II, Acute Physiology and Chronic Health Evaluation II; m-NUTRIC, Modified Nutrition Risk in Patients with Critical Illness.

**Table 2:** CatBoost model performance in training and internal test sets.

Dataset	Class	Precision	Recall	F1-Score	Support
Training	Dead	0.829	0.800	0.814	419
Training	Alive	0.720	0.758	0.738	285
Internal test	Dead	0.847	0.790	0.817	119
Internal test	Alive	0.615	0.702	0.656	57

Note: The positive class was defined as death.

**Table 3:** Comparison of the performance of the CatBoost model and logistic regression in the internal test set.

Model	Class	Precision	Recall	F1-Score	Support
CatBoost	Dead	0.847	0.790	0.817	119
CatBoost	Alive	0.615	0.702	0.656	57
Logistic Regression	Dead	0.838	0.739	0.786	119
Logistic Regression	Alive	0.563	0.702	0.625	57

Note: The positive class was defined as death.

**Table 4:** Comparison of the CatBoost and logistic regression models in the internal test set.

Metric	CatBoost	Logistic Regression	Δ
ROC-AUC	0.827	0.797	0.030
ROC-AUC 95% CI	(0.760–0.887)	(0.720–0.861)	—
Accuracy	0.761	0.727	0.034
Sensitivity (Dead)	0.790	0.739	0.051
Specificity (Alive)	0.702	0.702	0.000
F1-Score (Dead)	0.817	0.786	0.031
F1-Score (Alive)	0.656	0.625	0.031

Note: The positive class was defined as death.

**Table 5:** Summary of the performance metrics of the CatBoost model.

Performance Metric	Training Set	Internal Test Set
Sample size (n)	704	176
ROC-AUC (95% CI)	0.850 (0.822–0.879)	0.827 (0.760–0.887)
Overall Accuracy	0.783	0.761
Precision (dead class)	0.829	0.847
Recall/sensitivity (dead class)	0.800	0.790
F1 score (dead class)	0.814	0.817
Precision (Alive class)	0.720	0.615
Recall/Specificity (Alive class)	0.758	0.702
F1 score (Alive class)	0.738	0.656
PR-AUC (average precision)	—	0.909
Brier Score	—	0.186
Classification Threshold	0.482 (Youden J)	0.482 (frozen)

Note: The positive class was defined as death.

## Data Availability

The dataset is not publicly available due to institutional policy and ethical and privacy restrictions but can be made available through the corresponding author upon reasonable request.

## Supplementary Materials

The following supporting information can be downloaded at: https://www.mdpi.com/article/10.3390/jcm15062310/s1, Figure S1: Global feature importance based on mean absolute SHAP values; Figure S2: SHAP dependence plot for m-NUTRIC score, colored by patient age; Figure S3: SHAP dependence plot for age, colored by m-NUTRIC score; Figure S4: SHAP dependence plot for serum zinc concentration, colored by patient age.

## Abbreviations

AP	Average precision
APACHE II	Acute Physiology and Chronic Health Evaluation II
AUC	Area under the curve
CCI	Charlson Comorbidity Index
CI	Confidence interval
CPR	Cardiopulmonary resuscitation
DCA	Decision curve analysis
EMR	Electronic medical record
GCS	Glasgow Coma Scale
ICU	Intensive care unit
IHCA	In-hospital cardiac arrest
LR	Logistic regression
m-NUTRIC	Modified Nutrition Risk in Patients with Critical Illness
MICU	Medical intensive care unit
ML	Machine learning
PR	Precision–recall
ROC	Receiver operating characteristic
ROSC	Return of spontaneous circulation
SD	standard deviation
SHAP	SHapley Additive ExPlanations
SOFA	Sequential Organ Failure Assessment

## References

[1] Andersen L.W., Holmberg M.J., Berg K.M., Donnino M.W., Granfeldt A. (2019). In-Hospital Cardiac Arrest: A Review. JAMA.

[2] Girotra S., Nallamothu B.K., Spertus J.A., Li Y., Krumholz H.M., Chan P.S. (2012). Trends in survival after in-hospital cardiac arrest. N. Engl. J. Med..

[3] Stoltzfus J.C. (2011). Logistic regression: A brief primer. Acad. Emerg. Med..

[4] Christodoulou E., Ma J., Collins G.S., Steyerberg E.W., Verbakel J.Y., Van Calster B. (2019). A systematic review shows no performance benefit of machine learning over logistic regression for clinical prediction models. J. Clin. Epidemiol..

[5] Lapp L., Roper M., Kavanagh K., Bouamrane M.M., Schraag S. (2023). Dynamic prediction of patient outcomes in the intensive care unit: A scoping review of the state-of-the-art. J. Intensive Care Med..

[6] Rajkomar A., Dean J., Kohane I. (2019). Machine learning in medicine. N. Engl. J. Med..

[7] Salahuddin Z., Woodruff H.C., Chatterjee A., Lambin P. (2022). Transparency of deep neural networks for medical image analysis: A review of interpretability methods. Comput. Biol. Med..

[8] Safaei N., Safaei B., Seyedekrami S., Talafidaryani M., Masoud A., Wang S., Li Q., Moqri M. (2022). E-CatBoost: An efficient machine learning framework for predicting ICU mortality using the eICU collaborative research database. PLoS ONE.

[9] Lundberg S.M., Erion G., Chen H., DeGrave A., Prutkin J.M., Nair B., Katz R., Himmelfarb J., Bansal N., Lee S.-I. (2020). From local explanations to global understanding with explainable AI for trees. Nat. Mach. Intell..

[10] Rahman A., Hasan R.M., Agarwala R., Martin C., Day A.G., Heyland D.K. (2016). Identifying critically-ill patients who will benefit most from nutritional therapy: Further validation of the “Modified NUTRIC” Nutritional Risk Assessment Tool. Clin. Nutr..

[11] Lim S.L., Ling R.R., Lim O., Ueno R., Jones D., Low C., Ong M.E., Ridley E.J., Sundararajan K., Pilcher D. (2025). Obesity and nutrition risk in patients admitted to intensive care after cardiac arrest: A multicenter cohort study. Resuscitation.

[12] Frederiks P., Peetermans M., Wilmer A. (2024). Nutritional support in the cardiac intensive care unit. Eur. Heart J. Acute Cardiovasc. Care.

[13] de Man A.M.E., Amrein K., Casaer M.P., Dizdar O.S., van Zanten A.R.H., Gundogan K., Lepp L., Rezzi S., Shenkin A., Berger M.M. (2024). LLL 44-4: Micronutrients in acute disease and critical illness. Clin. Nutr. ESPEN.

[14] Liu B., Li M., Wang J., Zhang F., Wang F., Jin C., Li J., Wang Y., Sanderson T.H., Zhang R. (2024). Role of magnesium in cardiac arrest. Front. Nutr..

[15] Zhang Y.P., Wan Y.D., Sun T.W., Kan Q.C., Wang L.X. (2014). Association between vitamin D deficiency and mortality in critically ill adult patients: A meta-analysis of cohort studies. Crit. Care.

[16] Collins G.S., Dhiman P., Ma J., Schlussel M.M., Archer L., Van Calster B., E Harrell F., Martin G.P., Moons K.G.M., van Smeden M. (2024). Evaluation of clinical prediction models (Part 1): From development to external validation. BMJ.

[17] Friedrich S., Groll A., Ickstadt K., Kneib T., Pauly M., Rahnenführer J., Friede T. (2023). Regularization approaches in clinical biostatistics: Methods and applications. Stat. Methods Med. Res..

[18] Collins G.S., Moons K.G.M., Dhiman P., Riley R.D., Beam A.L., Van Calster B., Ghassemi M., Liu X., Reitsma J.B., Van Smede M. (2024). TRIPOD+AI statement: Updated guidance for reporting clinical prediction models that use regression or machine learning methods. BMJ.

[19] Yoo W., Jang H., Seong H., Kim S., Kim S.H., Jo E.J., Eom J.S., Lee K. (2024). Modified NutriC Score’s Ability to Predict Mortality in Patients Requiring Short-Term versus Prolonged Acute Mechanical Ventilation: A Retrospective Cohort Study. Ther. Adv. Respir. Dis..

[20] Li K., Shi Q., Liu S., Xie Y., Liu J. (2021). Predicting in-hospital mortality in patients with sepsis in the ICU using a gradient boosting decision tree. Medicine.

[21] Saito T., Rehmsmeier M. (2015). The precision-recall plot is more informative than the ROC plot for evaluating binary classifiers on imbalanced datasets. PLoS ONE.

[22] Allgaier J., Mulansky L., Draelos R.L., Pryss R. (2023). How does the model make predictions? A systematic literature review on the explainability power of machine learning in healthcare. Artif. Intell. Med..

[23] Koekkoek W.A.C., Hettinga K., de Vries J.H.M., van Zanten A.R.H. (2021). Micronutrient deficiency in critical illness. Clin. Nutr..

[24] Koekkoek K.W.A.C., van Zanten A.R.H. (2018). Nutrition in the ICU. Curr. Opin. Anaesthesiol..

[25] Varayil J.E., Bielinski S.J., Mundi M.S., Bonnes S.L., Salonen B.R., Hurt R.T. (2025). Artificial intelligence in clinical nutrition: Bridging data analytics and nutritional care. Curr. Nutr. Rep..

[26] Vickers A.J., van Calster B., Steyerberg E.W. (2019). A simple, step-by-step guide to interpreting decision curve analysis. Diagn. Progn. Res..

[27] Adams R., Henry K.E., Sridharan A., Soleimani H., Zhan A., Rawat N., Johnson L., Hager D.N., Cosgrove S.E., Markowski A. (2022). Prospective, multi-site study of patient outcomes after implementation of the TREWS machine learning-based early warning system for sepsis. Nat. Med..

[28] Sendak M.P., Ratliff W., Sarro D., Alderton E., Futoma J., Gao M., Nichols M., Revoir M., Yashar F., Miller C. (2020). Real-world integration of a sepsis deep learning technology into routine clinical care: Implementation study. JMIR Med. Inform..

[29] Wong A., Otles E., Donnelly J.P., Krumm A., McCullough J., DeTroyer-Cooley O., Pestrue J., Phillips M., Konye J., Penoza C. (2021). External validation of a widely implemented proprietary sepsis prediction model in hospitalized patients. JAMA Intern Med..

