# Supplementary material for: Early Risk Stratification for 30-Day Mortality After In-Hospital Cardiac Arrest: SHAP Interpretable CatBoost Model with m-NUTRIC and Micronutrient Biomarkers
Source: J Clin Med. 2026 Mar 18;15(6):2310. doi: 10.3390/jcm15062310 (PMC13027057; doi:10.3390/jcm15062310)
Supplement: Supplementary file 1 [file jcm-15-02310-s001.zip › jcm-4104829-supplementary.pdf]

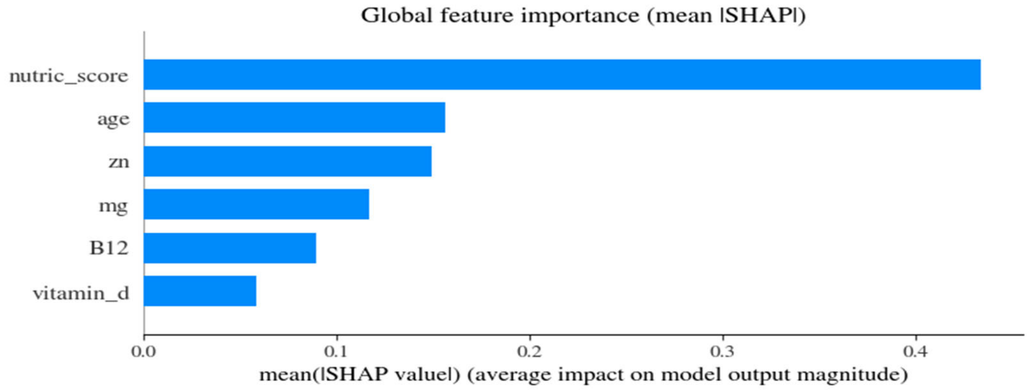

Figure S1. Global feature importance based on mean absolute SHAP values. NUTRIC score emerges as the dominant predictor with substantially higher mean |SHAP| value, followed by age, serum zinc, magnesium, vitamin B12, and vitamin D.

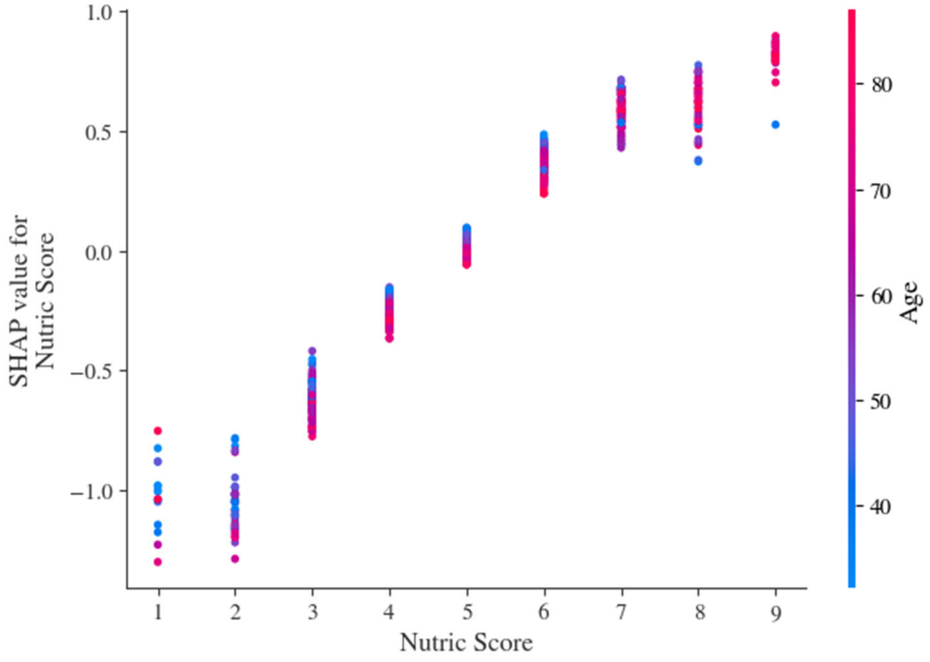

Figure S2. SHAP dependence plot for NUTRIC score, coloured by patient age. A strong monotonic positive relationship is evident: SHAP values increase progressively with higher NUTRIC scores, transitioning from negative contributions (protective) at low scores to substantial positive contributions (risk-increasing) at high scores. The relationship appears consistent across age groups.

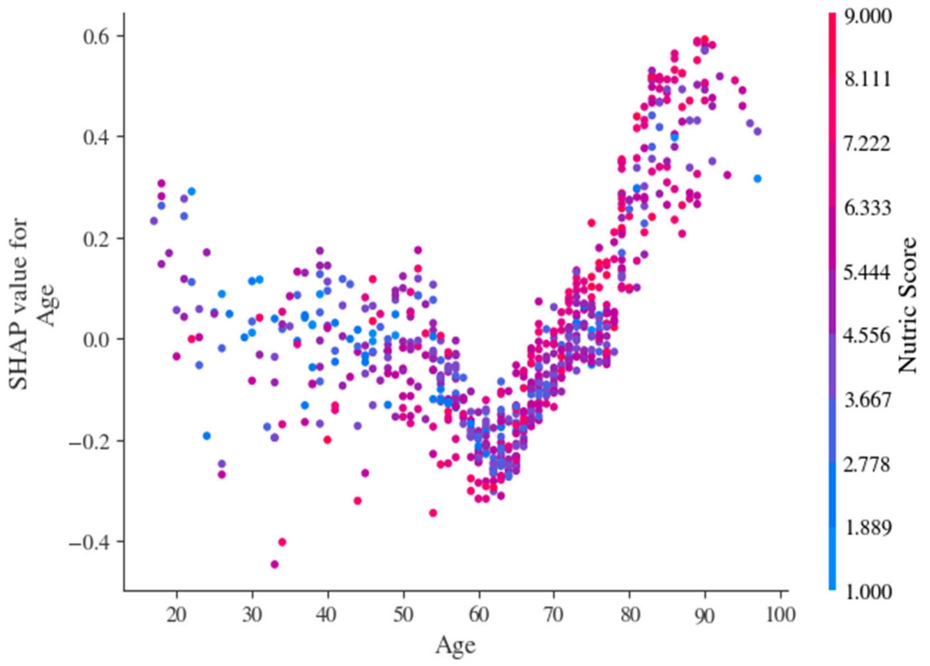

Figure S3. SHAP dependence plot for age, coloured by NUTRIC score. The relationship exhibits non-linearity: SHAP values remain relatively modest and slightly negative for younger patients (approximately 30–55 years), then increase more steeply beyond approximately 60 years of age. Patients with high NUTRIC scores (red) tend to have elevated SHAP values regardless of age.

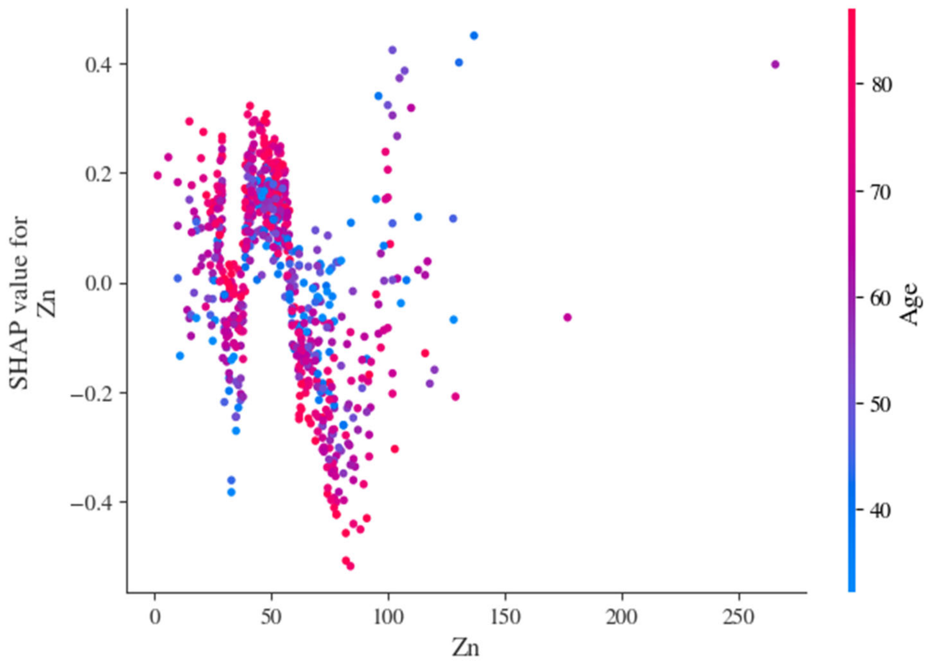

Figure S4. SHAP dependence plot for serum zinc concentration, coloured by patient age. Lower zinc levels are generally associated with positive SHAP values (increased predicted mortality), whilst higher concentrations correspond to negative SHAP values (protective effect). The effect appears more pronounced in older patients (red points).
